# Correspondence

**Published:** 1859-11

**Authors:** Edgar J. Waye


### Correspondence.

Sandusky, Ohio, Sept. 19, 1859.

Messrs. Editors:—Having read in the last Register the very modest and unbiased statements made by M. Loomis, before the Convention at Niagara, concerning the superior merits of his patent "Mineral plates," they being as he declares, the "ne plus ultra" of this department of dental science; "so entirely pure and clean in fulfilling its purpose, and fulfilling that purpose so well, and *never* wearing out or giving way." And that "having tested it in practical operation for six years, he knows not a single objection to be brought against it other than that it is artificial."

Unfortunately for the reputation of the "ne plus ultra" plate in this region, the talented inventor resides at too great a distance to be personally employed by the admirers of that style of work. Not that we are by any means destitute of that great blessing, Mineral plates in Northern Ohio, indeed, are quite common; but they seem to be of a kind which differs widely from that described as "fulfilling its purpose *so* well, and never wearing out or giving way." So much so indeed, that many of these plates persistently refuse to perform any useful purpose whatever. They can not be persuaded even to "stay in," to say nothing of the more desirable purposes of mastication.

Moreover, they are the victims of every accident. A blow or a slight fall breaks them, and when this occurs, the truth of his declaration "that where this plan is adopted, repairing will be abandoned in toto," will be felt in its full force and beauty; for repairing broken mineral plates is simply impossible. In this respect, I concede that this work has the advantage over all others; for while it spares the dentist the trouble of mending his broken work, it delights his pocket with the price of a second set; a peculiarity which will tend to elevate it, I doubt not, in the estimation of such of the profession as have had an unhappy exposure in that way.

Again, the mineral plate (what I have seen of it) is thicker than gold, continuous or Vulcanite; and it would seem must be so, to possess the requisite strength, consequently more clumsy and disagreeable in the mouth.

Now, I have no interest in any patent material whatever, save "Cheoplasty" (which I do not use, and wish I never had), and may, therefore, fairly be supposed as impartial at least as the inventor and manufacturer; and from all that I have seen and learned in regard to this kind of work, I consider it in most respects inferior to the Vulcanite. There are difficulties in its manufacture which do not exist in the latter, and when they are surmounted, it is no better. It is not every good practical dentist who can carve a tooth or a set of them artistically, though he might mount them on a plate unexceptionably. Again, mineral plate shrinks in baking, and shrinks unequally as the thickness of the plate is unequal, and this difficulty can not, I conceive, be entirely overcome, in which case it must be ground to fit—a tedious and difficult operation. With the Vulcanite, on the contrary, all that is necessary to secure a perfect and exquisite adaptation is to get a *perfect* impression; that obtained, the result is certain, and for the rest, its advantages, as compared with the mineral, are as follows:

1st. It is as light, with less thickness and greater strength;
2d. It is as cleanly;
3d. If broken, it may be repaired;
4th. It can be afforded as cheaply.

In short, it has all its advantages, without its defects.

If, as the inventor says, there are those "whose pecuniary interests are adverse to the introduction of his work," it is not, I opine, for the lack of skill to construct, neither is it the "clog of Letters Patent," but rather its inferiority to other methods and materials. Whenever it can be shown to be a better and cheaper work than any other, it will be the interest of every member of the profession to introduce it, and it will be done, and by none with greater alacrity and pleasure than the subscriber. EDGAR J. WAYE.

